# Severe Hemoperitoneum After Oocyte Retrieval Due to Ovarian Hemorrhage Possibly Caused by Violent Cough Under General Anesthesia With Propofol and Pentazocine: A Case Report and Literature Review

**DOI:** 10.7759/cureus.18724

**Published:** 2021-10-12

**Authors:** Chihiro Okoshi, Toshifumi Takahashi, Kuniaki Ota, Ryota Suganuma, Keiya Fujimori

**Affiliations:** 1 Department of Obstetrics and Gynecology, Fukushima Medical University, Fukushima, JPN; 2 Fukushima Medical Center for Children and Women, Fukushima Medical University, Fukushima, JPN

**Keywords:** oocyte retrieval, cough, hemoperitoneum, ovarian hemorrhage, anesthesia

## Abstract

Oocyte retrieval is an invasive procedure for patients undergoing assisted reproductive technology (ART). Complications regarding oocyte retrieval are rare; however, once they occur, patients often require hospitalization and, could in some cases, lead to life-threatening situations.

We report a case of severe hemoperitoneum due to ovarian hemorrhage after oocyte retrieval. A 39-year-old patient wanted to have a second child and received ART treatment because of severe male infertility. Oocyte aspiration was performed under intravenous anesthesia with propofol and pentazocine. She suddenly coughed and violently moved her body while the puncture needle was inside the ovary. After increasing the propofol dose, the coughing subsided and oocyte retrieval continued. Five hours following oocyte retrieval, she revisited the clinic with severe abdominal pain. Transvaginal ultrasonography revealed echogenic findings in the Douglas’ pouch; 300 mL of blood was aspirated by puncture of the Douglas pouch. She was transferred to our hospital because of suspected severe hemoperitoneum. Contrast-enhanced computed tomography revealed hematoma formation in the right ovary with a small amount of extravasation. Laparoscopic surgery revealed extensive hemoperitoneum due to right ovarian hemorrhage. The right ovary was enlarged to the size of a fist and contained a clot; the bleeding was due to a tear in the cortex of the ovary. The partially torn ovarian tissue was excised; hemostasis was performed using a bipolar device. The body movement caused by violent coughing during oocyte retrieval may have led to increased laceration at the ovarian puncture site.

Although cough during general anesthesia using propofol and pentazocine is very rare, physicians and staff should be aware of the high risk of intra-abdominal hemorrhage when such complications occur.

## Introduction

The use of assisted reproductive technology (ART) is increasing globally. Over 40 years have elapsed since the birth of the first child using the ART procedure of in vitro fertilization (IVF). Subsequently, there has been the development and advancement of various procedures, including controlled ovarian stimulation, methods of oocyte retrieval, insemination methods, embryo culture techniques, embryo transfer, and oocyte and embryo cryopreservation [1].

As a result, all ART procedures can be safely implemented at the outpatient clinic of specialized ART centers. The oocyte retrieval process is the only ART procedure that is invasive to patients. Complications associated with oocyte retrieval include vaginal wall bleeding, ovarian hemorrhage due to ovarian puncture, damage to the intra-abdominal organs, such as the intestine or colon, bladder, ureter, and large blood vessels, and pelvic infection [2-4]. These complications are rare; however, when they occur, patients often require hospitalization and such complications could in some cases lead to life-threatening situations [5].

General anesthesia is performed via inhalation or intravenously during oocyte retrieval to relieve pain and anxiety [6]. Complications during anesthesia, particularly during induction, include coughing and hiccups, which can induce body movements during oocyte retrieval and increase the risk of intra-abdominal organ damage due to semi-blind vaginal puncture into the abdominal cavity. Although complications such as coughing and hiccups under general intravenous anesthesia have been reported [7-9], there are no reports of such complications associated with anesthesia during oocyte retrieval.

We encountered a case of severe hemoperitoneum after oocyte retrieval due to ovarian hemorrhage, possibly caused by sudden coughing of the patient while under intravenous anesthesia during oocyte retrieval; laparoscopic hemostasis was required. We report the case and discuss available literature on complications during oocyte retrieval in patients who underwent ART treatment.

## Case presentation

The patient was a 39-year-old Japanese female, gravida 1 para 1. She wanted to have a second child. For the first pregnancy, the patient underwent ART treatment due to severe oligoasthenozoospermia of her husband. She received intracytoplasmic sperm injection (ICSI) followed by embryo cryopreservation. A successful pregnancy was established by vitrified-warmed embryo transfer, and the child was delivered vaginally. The pregnancy was uneventful and no problems were noted in the patient's medical history. The patient decided to undergo ICSI treatment at the same clinic at which she became pregnant previously. Follicle-stimulating hormone and human menopausal gonadotropin were used for controlled ovarian stimulation under a gonadotropin-releasing hormone (GnRH) agonist long protocol. Recombinant human chorionic gonadotropin (250 µg) (Ovidorel, Merck Biopharma, Tokyo, Japan) was administered 36 h before oocyte retrieval to trigger oocyte maturation. Oocyte aspiration was performed under intravenous anesthesia with propofol and pentazocine. Atropine sulfate (0.5 mg) was premeditated, and pentazocine (15 mg) was administered intravenously. Subsequently, propofol (50 mg) was administered rapidly, with additional 10-20 mg doses until loss of consciousness. The patient did not respond to verbal commands and had lost spontaneous movements. After thorough vaginal lavage with saline, the follicles in the ovaries were punctured through the vaginal wall using a 17-gauge oocyte retrieval needle under transvaginal ultrasound guidance. The patient suddenly coughed and violently moved her body whilst the puncture needle was inside the ovary. After deepening the anesthesia by increasing the dose of propofol, the coughing subsided and no body movements were observed; therefore, oocyte retrieval was continued and 11 oocytes were retrieved. The patient had no symptoms after the oocyte retrieval and went home after 2 h of bed rest. Five hours following oocyte retrieval, she returned to the clinic with severe abdominal pain. Transvaginal ultrasonography revealed echogenic findings in the Douglas pouch. Douglas’ pouch puncture was performed, and 300 mL of blood was aspirated. Bleeding from the right ovary due to oocyte aspiration was suspected, and transvaginal compression was attempted. However, even after compression of the right ovary, blood accumulated in the abdominal cavity. Although her hemoglobin and hematocrit levels before oocyte retrieval were 13.4 g/dL and 40.6%, respectively, after compression of the ovary, the hemoglobin and hematocrit levels had decreased to 10.8 g/dL and 40.0%, respectively. Based on a transvaginal ultrasound and blood examination findings, it was determined that the patient had persistent bleeding from the right ovary into the abdominal cavity, and the patient was transferred to our hospital.

**Figure 1:**
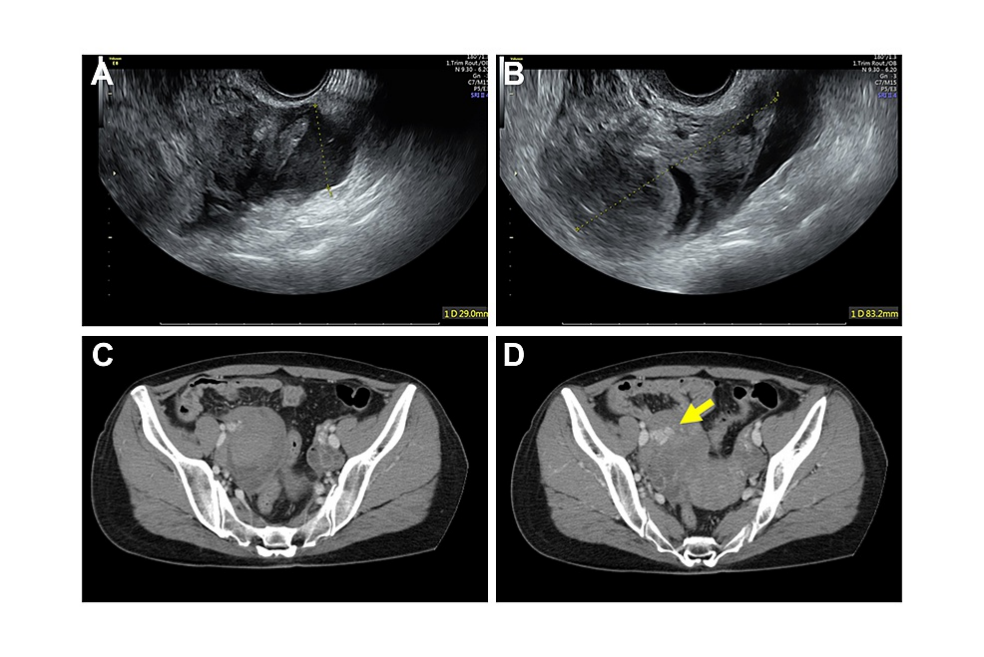
Photographs of transvaginal ultrasound and images of computed tomography (CT) of the patients with acute abdomen after oocyte retrieval at hospital transport. Echo-free space in the Douglas’s pouch (A) and swelling of the right adnexa (B) are observed. CT images of right adnexal hematoma (C) and contrast medium leaking from a blood vessel (D, arrow).

The patient’s characteristics were height: 161 cm, weight: 57 kg, and body mass index (BMI): 22 kg/m^2^. On arrival at our hospital, her blood pressure was 133/89 mmHg, pulse rate was 85/min, and shock index was 0.63. Her abdomen was flat, with tenderness and rebound pain. Internal examination was not performed because of severe pain. Transvaginal ultrasonography revealed an echogenic free space of 12.5 mm in the Douglas pouch and an enlarged right ovary with a size of 65 mm (Figure 1A, 1B). These findings suggested hemoperitoneum due to bleeding from the right ovary. Contrast-enhanced computed tomography revealed hematoma formation in the right ovary with a small amount of extravasation (Figure 1C, 1D). The diagnosis of hemoperitoneum from the right ovary due to oocyte retrieval was made. Since the vital signs were maintained and the pain was controlled, we managed the patient conservatively. After 2 h, her blood pressure was 150/82 mmHg and pulse rate was 101/min, the size of the echo-free space in the Douglas pouch and the size of the ovary were enlarged to 29 and 80 mm, respectively. Emergency laparoscopy was performed to ascertain the location of the bleed and to stop the bleeding.

**Figure 2:**
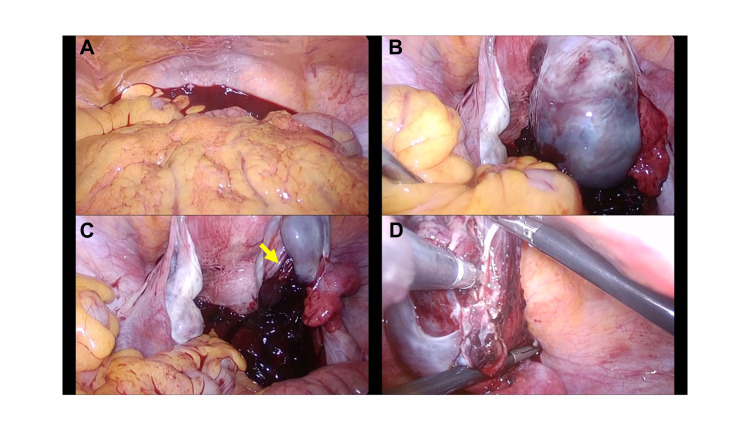
Photographs of laparoscopic findings in a patient with acute abdomen after oocyte retrieval. Hemoperitoneum (A) and right ovarian hematoma (B) are observed. Clotting mass from laceration of right ovary (C, arrow) and hemostatic coagulation using bipolar forceps (D).

Laparoscopic surgery was performed using four trocar ports with the patient under general anesthesia. A 12-mm trocar was inserted at the umbilicus under direct vision, and CO2 gas was insufflated to an intra-abdominal pressure of less than 10 mmHg. Three 5-mm trocars were placed in the lower abdomen on the right and left sides and in the middle. Laparoscopic findings revealed massive hemoperitoneum without intra-abdominal adhesions (Figure 2A). When the clot was removed, bleeding was confirmed in the right ovary. The right ovary was enlarged to the size of a fist, and hematoma was also observed inside the ovary (Figure 2B). When the hematoma was removed and the right ovary was examined in detail, the cortical region of the ovary was torn, and continuous bleeding was observed (Figure 2C). The partially torn ovarian tissue was excised, and the bleeding was coagulated and stopped using a bipolar device (Figure 2D). There were no abnormal findings in the left ovary. A drain was placed in the Douglas pouch, and the surgery was completed. The operative time was 57 min, and the estimated amount of blood lost was 200 mL. Postoperatively, there was no progression of anemia, and no blood transfusion was required. The patient had a favorable postoperative course and was discharged on the fourth following surgery. Histopathological examination revealed a corpus luteal hemorrhage in the excised ovarian tissue.

## Discussion

We encountered a rare case of severe hemoperitoneum after oocyte retrieval due to tearing of the ovary caused by violent coughing under general anesthesia with propofol and pentazocine.

Oocyte retrieval is the only invasive procedure undertaken during ART. Currently, in most cases, ovarian puncture is performed through the vaginal wall under transvaginal ultrasonography guidance. Complications of oocyte retrieval include ovarian hemorrhage, damage to organs such as the uterus, bladder, intestinal tract, and large blood vessels [10], and pelvic infection. The frequency of hemoperitoneum after oocyte retrieval has been reported to be 0.06-0.35% [10, 11]. On the other hand, even when oocyte retrieval is achieved without adverse events, the amount of intra-abdominal bleeding at 24 h was reported as 232 ± 130 mL (mean ± standard deviation), and hospitalization should be considered if intra-abdominal bleeding of 500 mL or more is suspected [12].

To date, 13 case reports or case series studies have reported the need for surgical hemostasis for intra-abdominal bleeding after oocyte retrieval, and clinical data were available for 34 patients, including our case [2-5, 13-21]. The clinical information in these cases is summarized in Table 1. The median patient age was 32 years (n=25, range: 20-42 years), the median BMI was 20.0 kg/m^2^ (n=16, range: 17.8-27.6), and 85.0% (17/20) of the patients had no history of delivery. The number of patients with normal BMI (18.5-24.9 kg/m^2^) was 14 out of 16 cases. The percentage of patients with thrombophilia or who received anticoagulant therapy was 33.3% (7/21). The number of patients using controlled ovarian stimulation was 27 out of 28. The median number of retrieved oocytes was 13 (n=27, range: 0-34). General anesthesia or intravenous anesthesia was used in all 21 cases in which the method of anesthesia during oocyte retrieval was described. The top three symptoms that prompted the detection of intra-abdominal bleeding were abdominal pain, nausea or vomiting, and dizziness or syncope. The number and percentage of cases were abdominal pain: 19/28 (67.9%); nausea or vomiting: 17/28 (60.7%); and dizziness or syncope: 7/28 (25%). The median time from oocyte retrieval to the onset of symptoms was 10.0 h (n=19, range: 2 hrs-10 days). Of the 25 cases in which the gauge of the puncture needle used for oocyte retrieval was specified; a 17-gauge, 18-gauge, and 19-gauge needle was used in 22 cases (88%), one case (4%), and two cases (8%), respectively. With regard to the method of surgical procedure, 14/34 (41.2%) could be completed solely by laparoscopy, 4/34 (11.8%) were converted from laparoscopy to laparotomy, 15/34 (44.1%) were performed by laparotomy, and 1/34 (2.9%) underwent uterine artery embolization. The median estimated blood loss was 1000 mL (n=23, range: 25-25 mL), and blood transfusion was performed in 18 of 32 cases (56.3%). The bleeding originated in the ovary in 30 cases (n=33, 90.9%), and one of the remaining three cases was hemoperitoneum from the infundibulo-pelvic ligament, whereas the other two cases were retroperitoneal hematomas due to bleeding from the retroperitoneal vein or artery.

**Table 1:** Cases of treatment for intra-abdominal bleeding after oocyte retrieval during ART treatment. *Two episodes in the same case. BMI, body mass index; COS, controlled ovarian stimulation; GnRHa, gonadotropin-releasing hormone agonist; GnRHant, gonadotropin-releasing hormone antagonist; LMWH, low-molecular-weight heparin; NA, not available; SO, salpingo-oophorectomy; UAE, uterine artery embolization

Case no.	First author (year) [reference]	Case report or case series	Age (years)	BMI (kg/m^2^)	Parity	Thrombophilia or anti-coagulant therapy	Previous oocyte aspiration	Ovarian stimulation	No. of retrieved oocytes	Needle size (gauge)	Anesthesia	Symptoms	Time from oocyte retrieval to onset	Intervention	Bleeding volume /blood transfusion	Bleeding origin
1	Azem et al. (2000) [13]	Case report	42	NA	0	No	Yes	COS	7	NA	NA	Vomit, abdominal pain	10 h	Laparoscopy and laparotomy, metal clipping	NA/yes	Mid sacral vein, retroperitoneal hematoma
2	Battaglia et al. (2001) [14]	Case report	34	NA	0	Factor XI decrease	No	COS (GnRHa)	9	18	NA	Syncope	3 h	Laparotomy, partial ovarian resection	250 mL/NA	Ovary
3	El-Shawarby et al. (2004) [15]	Case report	37	NA	0	Essential thrombosis, low dose aspirin	No	COS (GnRHant)	6	NA	Local and intravenous	Nausea, abdominal pain	28 h	Laparotomy, SO	3000 mL/yes	Ovary
4*	Moayeri et al. (2007) [16]	Case report	34	NA	0	Von Willebrand disease	No (1^st^) Yes (2^nd^)	NA	NA	NA	NA	Abdominal distention, scapular pain (1^st^), syncope (2^nd^)	10 h (1^st^), NA (2^nd^)	Laparoscopy, hemostasis (1^st^ and 2^nd^)	2200 mL/no (1^st^), 1500 mL/yes (2^nd^)	Ovary (both times)
5–9	Bodri et al. (2008) [3]	Case series (n = 5)	NA	NA	NA	NA	NA	COS (GnRHa, GnRHant) (n = 5)	8–23	17 (n = 5)	Intravenous (propofol and alfentanil) and inhalatory (n = 5)	NA	NA	Laparoscopy, hemostasis (n = 5)	NA/no (n = 5)	Ovary (n = 5)
10	Bandyopadhyay et al. (2010) [17]	Case report	36	NA	0	No	No	NA	NA	NA	Intravenous (propofol and alfentanil)	Abdominal discomfort, dizziness	2 h	Laparoscopy, hemostasis with suture	Over 1000 mL/no	Ovary
11–17	Liberty et al. (2010) [18]	Case series (n = 7)	20–41	19–21	0–3	Thalassemia (n = 1), no (n = 6)	NA (n = 7)	COS (GnRHa) (n = 6), (GnRHa, GnRHant) (n = 1)	11–34	17 (n = 7)	General (n = 7)	Abdominal pain (n = 4), nausea (n = 2), vomiting (n = 4), severe weakness (n = 3), fainting (n = 1), dizziness (n = 3)	5–18 h	Laparotomy and hemostasis (n = 6), laparotomy and hemostasis with suture (n = 1)	NA/yes (n = 7)	Ovary (tearing) (n = 6), ovary (n = 1)
18–22	Zhen et al. (2010) [19]	Case series (n = 5)	26–34	17.8–21.1	0 (n = 3), NA (n = 2)	No (n = 5)	Yes (n = 2), NA (n = 3)	COS (GnRHa) (n = 5)	7–18	17 (n = 5)	Intravenous (n = 2), NA (n = 3)	Abdominal pain (n = 4), nausea (n = 3), vomiting (n = 1), scapular pain (n = 1)	4 h–10 days	Laparotomy, hemostasis (n = 3), laparotomy, hemostasis with suture (n = 2)	1800 mL/no (n = 1), NA/yes (n = 1), NA/no (n = 3)	Ovary (n = 5)
23	Kart et al. (2011) [5]	Case report	40	NA	0	Factor VIII deficiency	Yes	NA	0	17	General	Abdominal pain, vomiting, vaginal bleeding	10 days	UAE	NA/Yes	NA
24–27	Aragona et al. (2011) [2]	Case series (n = 4)	NA (n = 4)	NA (n = 4)	NA (n = 4)	NA (n = 4)	NA (n = 4)	Natural cycle (n = 1), COS (GnRHa) (n = 1)	NA (n = 4)	NA (n = 4)	NA (n = 4)	Abdominal pain (n = 2), circulatory instability (n = 1), NA (n = 1)	2–12 h	Laparoscopy, hemostasis (n = 3), laparotomy, partial ovarian resection (n = 1)	750–1000 mL/no (n = 4)	Ovary (n = 4)
28–29	Mashiach et al. (2013) [20]	Case series (n = 2)	32 and 37	NA (n = 2)	NA (n = 2)	Essential thrombocytosis/LMWH and myeloproliferative disorder/LMWH	No (n = 2)	COS (GnRHant) and COS (GnRHa)	3 and 0	19 (n = 2)	NA (n = 2)	Abdominal pain, shoulder pain, abdominal bloating, tenesmus and abdominal pain, dyspnea	2 days and 3 days	Laparoscopy, hemostasis (n = 2)	2500 mL/yes and 2500 mL/yes	Infundibulo-pelvic ligament and ovary (tearing)
30–32	Nouri et al. (2014) [4]	Case series (n = 3)	25–32	18.6–27.6	0 (n = 2), 1 (n = 1)	NA (n = 3)	NA (n = 3)	COS (GnRHant) (n = 3)	10–19	17 (n = 3)	Intravenous (n = 3)	Abdominal pain (n = 3)	8–24 h	Laparoscopy and laparotomy, SO (n = 1), laparoscopy and laparotomy, hemostasis (n = 1), laparoscopy, hemostasis (n = 1)	NA/yes (n = 2), NA/NA (n = 1)	Ovary (n = 3)
33	Bolster et al. (2014) [21]	Case report	36	NA	NA	NA	Yes	NA	NA	NA	NA	Headache, collapse	4 days	Laparotomy, UAE	NA/yes	Pseudoaneurysm of the obturator artery, retroperitoneal hematoma
34	Okoshi et al. (present case)	Case report	39	22.0	1	No	Yes	COS (GnRHa)	11	17	Intravenous (propofol and pentazocine)	Abdominal pain	8 h	Laparoscopy, hemostasis with suture	500 mL/no	Ovary (tearing)

Several risk factors for severe hemoperitoneum after oocyte retrieval have been reported. Liberty et al. reported that lean-type polycystic ovary syndrome (PCOS) might be a risk factor for severe hemoperitoneum after oocyte retrieval. They reported that of 3241 oocyte retrievals, severe hemoperitoneum occurred in seven patients, four of whom were diagnosed with PCOS with a BMI of 19-21 kg/m^2^. Patients with lean-type PCOS exhibited more follicular growth and higher peak estrogen levels, which could be due to the fragility of the ovaries themselves [18]. Kuroda et al. [11] reported on the risk factors for severe hemoperitoneum based on observations from 1,435,108 oocyte retrievals during ART treatment. The highest risk factors for severe hemoperitoneum were the use of a GnRH agonist protocol and clinical pregnancy following fresh embryo transfer. Other risk factors for hematoperitoneum include previous ovarian surgery, predisposition to bleeding, a large number of oocytes retrieved, and a previous history of oocyte retrieval [4, 10]. In this case, the patient had a normal BMI, with no history of PCOS, ovarian surgery, or thrombophilia; however, previous oocyte retrieval and the use of a GnRH agonist long protocol were risk factors.

To prevent severe hemoperitoneum after oocyte retrieval, it is important to consider the risk factors mentioned above. Based on previous reports, patients with lean-type PCOS may benefit from controlled ovarian stimulation with a GnRH antagonist or mild ovarian stimulation, rather than stimulation with a GnRH agonist. Although no studies have examined whether the size of the oocyte aspiration needle is a risk for severe hemoperitoneum after oocyte retrieval, in our literature review, a 17-gauge needle was used in 88% of cases with severe hemoperitoneum after oocyte retrieval; therefore, the use of a thinner oocyte aspiration needle should be considered during oocyte retrieval.

The type of anesthesia to be used during oocyte retrieval is selected based on the number of oocytes expected to be retrieved, the patient's anxiety about pain, and the circumstances at the ART facility. If the number of oocytes to be retrieved is below five, the procedure can be performed with local anesthesia of the vaginal wall. General anesthesia applied intravenously is used in most ART facilities [6]. Anesthetics are known to be transported into the follicular fluid, and in vitro experiments have revealed that anesthetics can affect oocytes [22]. However, clinical data have shown that anesthetics used during oocyte retrieval do not affect fertilization rates, embryonic development, or pregnancy [23]. Anesthesia during oocyte retrieval contributes to an increase in the number of oocytes retrieved by eliminating the patient's anxiety and pain with sufficient depth of anesthesia.

There are few published reports on anesthetic complications during oocyte retrieval. Most anesthetic complications during egg retrieval involve nausea and vomiting [24], and there are no reports of coughing causing body movements. In this case, the body movement caused by violent coughing during oocyte retrieval may have increased laceration at the ovarian puncture site. There are few reports of cough reflex due to propofol, and it is considered a rare side effect. Case reports involving severe cough attacks during propofol anesthesia have been reported [7, 8]. However, pentazocine is an analgesic to suppress coughing during the use of narcotics such as fentanyl [25]. In this case, the cause of the coughing during oocyte retrieval is unknown, but the coughing attack may have been provoked by propofol, or the analgesia with pentazocine may have been insufficient and the patient may have been awakened by pain. In this case, propofol was used in anesthesia by rapid administration followed by additional boluses to maintain an adequate depth of anesthesia; however, no infusion pump or other device was used to monitor the depth of anesthesia or the blood levels during anesthesia. It may be difficult to maintain the depth of anesthesia with additional bolus doses, and the amount of pentazocine used for analgesia may have been insufficient to induce coughing and body movement due to pain.

## Conclusions

In conclusion, the oocyte retrieval procedure is the only invasive procedure in ART treatment. Adequate anesthesia is of great importance not only for pain relief but also to ensure optimal surgical conditions and no patient’s movements. Physicians and staff should be aware of the high risk of intra-abdominal hemorrhage when such complications occur.
